# Integrated human/SARS-CoV-2 metabolic models present novel treatment strategies against COVID-19

**DOI:** 10.26508/lsa.202000954

**Published:** 2021-08-05

**Authors:** Bridget P Bannerman, Jorge Júlvez, Alexandru Oarga, Tom L Blundell, Pablo Moreno, R Andres Floto

**Affiliations:** 1 Molecular Immunity Unit, Department of Medicine, University of Cambridge, Cambridge, UK; 2 Department of Biochemistry, University of Cambridge, Cambridge, UK; 3 Department of Computer Science and Systems Engineering, University of Zaragoza, Zaragoza, Spain; 4 EMBL-EBI, European Bioinformatics Institute, Hinxton, UK; 5 The Center for Research and Interdisciplinarity, Paris, France

## Abstract

The coronavirus disease 2019 (COVID-19) pandemic caused by the new coronavirus (SARS-CoV-2) is currently responsible for more than 3 million deaths in 219 countries across the world and with more than 140 million cases. The absence of FDA-approved drugs against SARS-CoV-2 has highlighted an urgent need to design new drugs. We developed an integrated model of the human cell and SARS-CoV-2 to provide insight into the virus’ pathogenic mechanism and support current therapeutic strategies. We show the biochemical reactions required for the growth and general maintenance of the human cell, first, in its healthy state. We then demonstrate how the entry of SARS-CoV-2 into the human cell causes biochemical and structural changes, leading to a change of cell functions or cell death. A new computational method that predicts 20 unique reactions as drug targets from our models and provides a platform for future studies on viral entry inhibition, immune regulation, and drug optimisation strategies. The model is available in BioModels (https://www.ebi.ac.uk/biomodels/MODEL2007210001) and the software tool, *findCPcli*, that implements the computational method is available at https://github.com/findCP/findCPcli.

## Abstract (precis)

Integrated human/SARS-CoV-2 metabolic models present novel treatment strategies against COVID-19 and provide insights into viral entry inhibition, immune regulation, and drug optimisation strategies.

## Introduction

SARS-COV-2, the causative agent of COVID-19, belongs to a group of viruses commonly known as β-coronavirus. This class of viruses is responsible for mild-to-fatal respiratory tract infections in animals and birds. Whereas the common cold is more commonly associated with the mild forms of the disease, the previous MERS and SARS-2002 infections and, currently, COVID-19 belong to the group of fatal diseases. The genome of the virus responsible for the ongoing COVID-19 disease, SARS-CoV-2, has ∼80% sequence identity to SARS-CoV and is 96% identical at the whole-genome level to a bat coronavirus (Zhou et al, 2020). SARS-CoV-2 affects the lower respiratory tract cells and the upper cells in the pharyngeal region (Chen et al, 2020; Huang et al, 2020), and the viral infections range from asymptomatic, mild, moderate, and severe cases. Previous studies in China show that 86% of cases of infection and the contagiousness of the virus were undocumented before travel restrictions were imposed (Li et al, 2020). In addition, the interim results from the Solidarity international clinical trial conducted by the World Health Organization confirmed that only corticosteroids are effective against severe and critical cases of COVID-19. The report shows little or no benefit from the other four treatments evaluated (remdesivir, hydroxychloroquine, lopinavir/ritonavir, and interferon) on overall mortality against COVID-19 (Dyer, 2020; Pan et al, 2021). Therefore, there are still many factors to unravel regarding the stages of infection and transmissibility patterns of the virus to achieve good treatment management strategies. Studies in France demonstrate the transmission potential of asymptomatic persons and suggest varying dynamics of transmission in children (Danis et al, 2020). The human angiotensin-converting enzyme 2 (human-ACE-2 protein) has been identified as the cell receptor for both the SARS-2002 virus and SARS-CoV-2. The ACE-2 enzyme, which has the primary function of controlling blood pressure, is usually found in the epithelial cells of the heart, lungs, kidneys, and intestine (Donoghue et al, 2000; Hamming et al, 2004; Liu et al, 2020).

**Figure 1.**
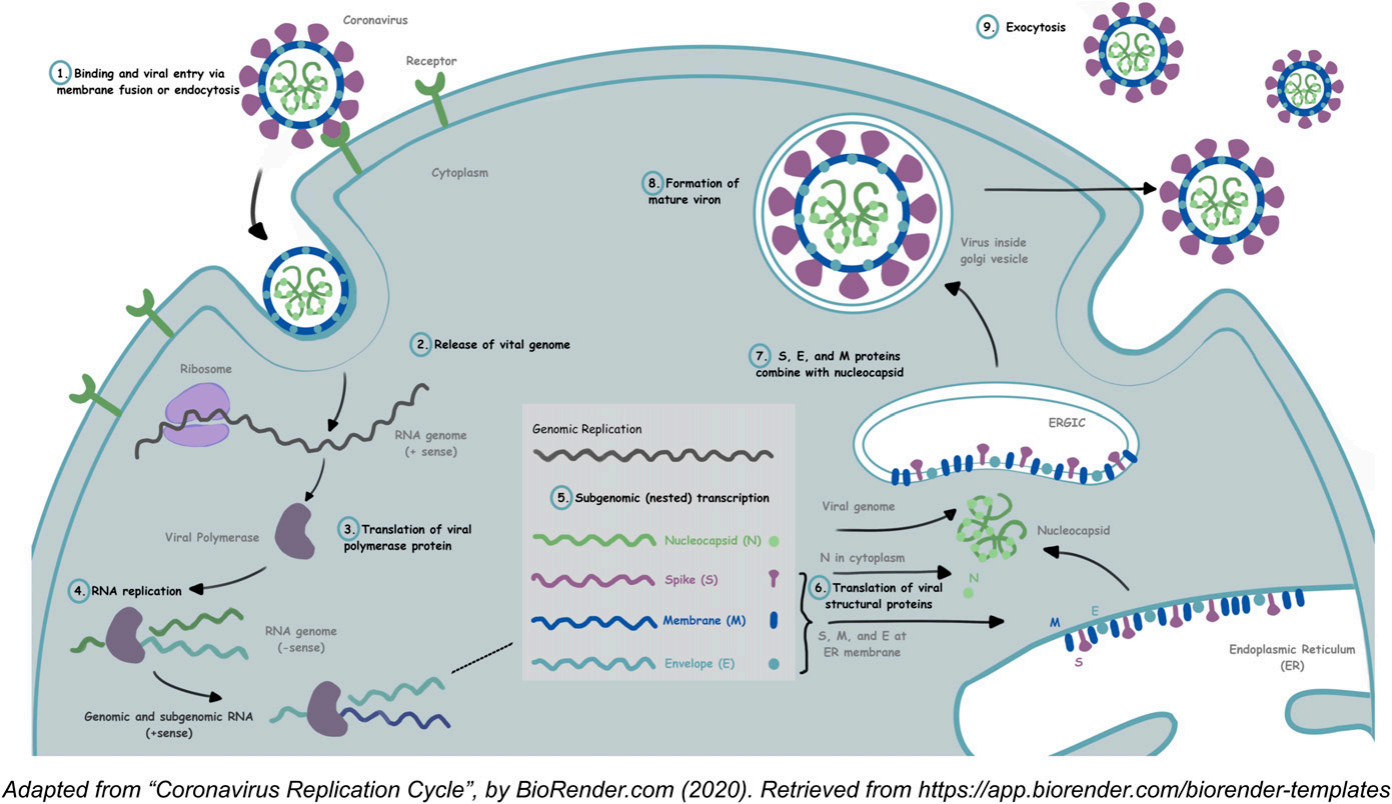
The mechanism of replication of SARS-CoV-2 in the human cell.

The mechanism of replication of SARS-CoV-2 in the human cell involves an initial binding and attachment of the spike (S) glycoprotein to the angiotensin-converting enzyme 2 (ACE2) receptor of its host. During endocytosis, the virus’s genetic material is injected into the host cell, where it loses its protective envelope (Fig 1). The subsequent assembly and maturation of viral proteins lead to cell death and proliferation of the virus within the human body.

The lack of FDA-approved drugs against COVID-19, coupled with the difficulties encountered globally in containing the virus, prompted the WHO to declare the outbreak a pandemic in March 2020. This has led to intensified efforts around the world to fight this disease. Previous studies in drug target identification against viral diseases such as Zika, chikungunya, and dengue by Aller et al, (2018) introduced a system of integrating the host’s macrophage and viral metabolic networks to predict a set of host reactions which, when constrained, can inhibit viral production. A recent study by Renz et al, (2020) demonstrates a similar approach and predicts drug targets against SARS-CoV-2. Targets of known antiviral drugs predicted from both studies, using a macrophage metabolic model (Bordbar et al, 2010) demonstrate the applicability of the integrated human/virus metabolic modelling in drug target identification. The alveolar macrophage host model (Bordbar et al, 2010) used to illustrate the metabolic interactions between multiple organisms was constructed from the *Homo sapiens* Recon 1 model (Duarte et al, 2007) based on previously built algorithms. This was followed by a set of manual curation processes to construct the context-specific alveolar macrophage host model (Bordbar et al, 2010). Similarly, Wang et al (2012) developed several draft context-specific models using the Human Recon 1 model.

We have built on these approaches by developing an integrated epithelial cell/SARS-CoV-2 metabolic model and used a combination of structural and dynamical analyses to assess the model and make predictions. We have applied the recently released community standards to facilitate our development of a standardised model for the systems biology international community and used the MEMOTE quality control software to assess and compare our model with previously developed GEMS (Carey et al, 2020, Lieven et al, 2020). We have designed a novel computational method and developed a software tool (*findCPcli*) to carry out such analyses and to predict drug targets. Both the model and the tool have been uploaded to public repositories (see the Data Availability section). We have also performed a comparative analysis of our model and other previously generated cell-type models.

## Results

### Construction of the human airway epithelial cell and the SARS-CoV-2 virus

We constructed an integrated genome-scale metabolic model (GEM) of the human airway epithelial cell with the SARS-CoV-2 virus using the methods described in Aller et al (2018) and Renz et al (2020). We used a previous draft reconstruction of the epithelial cell (Wang et al, 2012) and improved on the context-specificity of the GEM with the addition and simulation of data from the gene expression omnibus on COBRApy (Ebrahim et al, 2013). We performed further refinement from literature and by using the human metabolic networks in the HumanCyc database (Trupp et al, 2010) and Recon3D (Brunk et al, 2018). Model checks were done with MEMOTE, and refinements were performed with MetaNetX 4.2, BiGG, ChEBI, MetaCyc, and PubChem databases (Caspi et al, 2014; Hastings et al, 2016; Norsigian et al, 2020; Kim et al, 2021; Moretti et al, 2021). The new GEM contains 4,660 genes, 3,614 reactions, and 4,052 metabolites and conforms to the minimum standardised content for a newly published GEM based on recently published community standards (Carey et al, 2020); 100% of the metabolites in (*i*HsaEC21) have a human-readable descriptive name, 100% have an inchi key, 100% of metabolite annotation conformity with the BiGG database and in MetaNetX, Kyoto Encyclopedia of Genes and Genomes (KEGG), ChEBI, ModelSEED, HMDb, or MetaCyc (Caspi et al, 2014; Hastings et al, 2016; Wishart et al, 2018; Norsigian et al, 2020; Kanehisa et al, 2021; Kim et al, 2021; Moretti et al, 2021; Seaver et al, 2021); 100% of the metabolites have a charge and chemical formula with a charge balance of 75.3% (Supplemental Data 1). In addition, 97% of the reactions have a human-readable descriptive name, 100% of reactions conform with the BiGG database and as well as in MetaNetX, KEGG, ChEBI, ModelSEED, HMDb, or MetaCyc. The Gene IDs in the model are consistent with gene annotations in Uniprot with 92.5% of reactions having Gene-Protein-Reaction associations and 71.9% having the Enzyme Classification code.

Supplemental Data 1. MEMOTE results of the integrated human airway epithelial model and SARS-CoV-2 (*i*HsaEC21_SARS-CoV-2).

### Comparative analysis of integrated models of infected human epithelial cell and the macrophage cell with SARS-CoV-2

For our study, we performed a comparative analysis of the essential and unique reactions needed for the viability of the virus in the epithelial cell/SARS-CoV-2 integrated model and the GEM constructed by Renz et al (2020). Our results show how the virus heightens its virulence mechanisms by modifying the host’s defences within different cell compartments. Consequently, we suggest treatment regimens based on different stages of viral infection and replication.

### Host-dependent metabolic pathways

**Figure 2.**
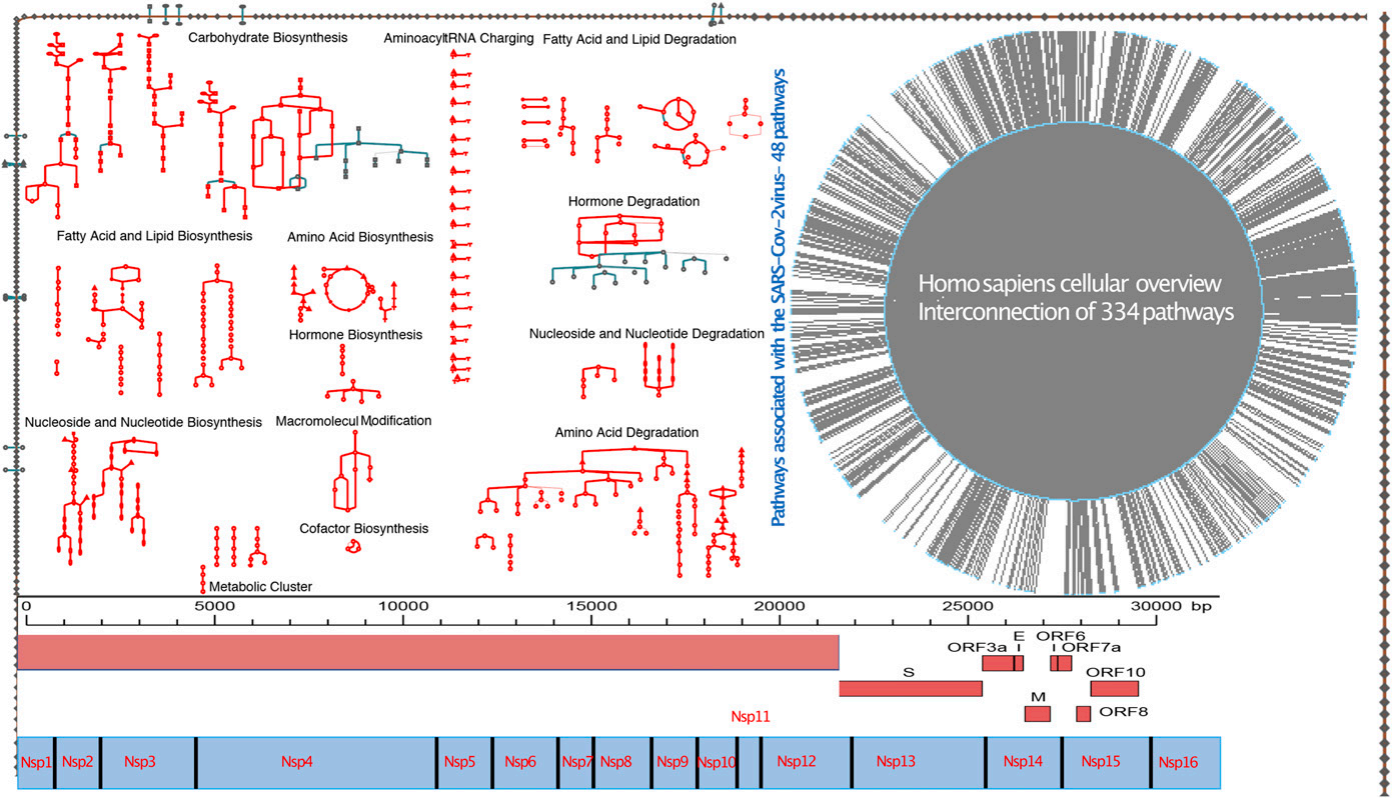
SARS-CoV-2 viral genome and host-dependent metabolic pathways.

We initially demonstrated the biochemical requirements for the maintenance of the human airway epithelial and macrophage cells and used the integrated models to show the essential host reactions needed for the survival and viability of SARS-CoV-2 within the host’s cell compartments. We have mapped the experimentally characterized human/SARS-CoV-2 protein–protein interaction data from Gordon et al (2020) on the in silico virus-integrated human macrophage and epithelial cells. Of the 334 metabolic pathways in the human metabolic network, 48 pathways including the biosynthesis and degradation pathways of amino acids, fatty acids, carbohydrates, amines, cofactors, and core components of the central mRNA metabolism are hijacked by the virus for its survival strategies (Fig 2).

The 48 metabolic pathways that were mapped to the protein–protein interaction network produced by Gordon et al (2020) are referred to as PPi-pathway intersection nodes in this article (Table 1). These include cysteine, methionine, and selenocysteine amino acid biosynthetic pathways; C20 prostanoid hormone biosynthetic pathways; and vitamin D3 and vitamin K epoxide cycle. The degradation pathways identified include the lysine, tryptophan, methionine, fatty acid degradation, ceramide, and sphingolipid recycling pathways; phospholipases degradation; and amine and heme degradation (Table 1).

**Table 1.** List of bottleneck and essential enzymes on the PPi-pathway intersection nodes.

Class	Pathway	Sub-pathway	Human gene	Sars gene	Macrophage	Epithelial cell
Biosynthesis	Amino acids	L-selenocysteine biosynthesis	*SEPSECS*	Nsp8	Y	N
Biosynthesis	Amino acids	Cysteine, and methionine	*MAT2B*	Nsp9	Y	Y
Biosynthesis	Fatty acids	Fatty acid and long fatty acid biosynthesis	*SLC27A2*	Nsp2	Y	Y
Biosynthesis	Fatty acids	Fatty acid and long fatty acid biosynthesis	*ACSL3*	Nsp7	Y	N
Biosynthesis	Fatty acids	Stearate biosynthesis	*SLC27A2*	Nsp2	Y	Y
Biosynthesis	Fatty acids	Stearate biosynthesis	*ACSL3*	Nsp7	Y	Y
Biosynthesis	Carbohydrate biosynthesis	Glycan and oligosaccharide biosynthesis	*ALG11*	Nsp4	Y	Y
Biosynthesis	Carbohydrate biosynthesis	Glycan	*HS2ST1*	Orf8a	Y	Y
Biosynthesis	Carbohydrate biosynthesis	Glycan	*MOGS*	Nsp7	Y	Y
Biosynthesis	Carbohydrate biosynthesis	Glycan and oligosaccharide biosynthesis	*ALG5*	ORF3a	Y	Y
Biosynthesis	Carbohydrate biosynthesis	Glycan, oligosaccharide, and glycosaminoglycan biosynthesis	*CHPF*	ORF8a	Y	Y
Biosynthesis	Carbohydrate biosynthesis	Glycan, oligosaccharide, and glycosaminoglycan biosynthesis	*HS6ST2*	ORF8a	Y	Y
Biosynthesis	Cofactors	Vitamin D3 biosynthesis	*POR*	Nsp2	Y	Y
Biosynthesis	Cofactors	Vitamin K epoxide cycle	*GGCX*	Main protease	N	Y
Biosynthesis	Hormones	C20 prostanoid biosynthesis	*PTGES2*	Nsp7	Y	Y
Biosynthesis	tRNA charging	tRNA charging	*TARS2*	Main protease	Y	Y
Degradation	Amino acids	L-lysine degradation	*AASS*	Main protease	N	Y
Degradation	Amino acids	L-tryptophan degradation	*POR*	Nsp2	Y	Y
Degradation	Amino acids	L-methionine degradation	*MAT2B*	Main protease	Y	Y
Degradation	Fatty acids	Ceramide and sphingolipid recycling	*SLC27A2*	Nsp2	Y	Y
Degradation	Fatty acids	Ceramide and sphingolipid recycling	*POR*	Nsp2	Y	Y
Degradation	Fatty acids	Ceramide and sphingolipid recycling	*ACSL3*	Nsp7	Y	Y
Degradation	Fatty acids	Phospholipases	*PLD3*	ORF8a	Y	Y
Degradation	Amine degradation	Dopamine degradation	*COMT*	Nsp7	Y	Y
Degradation	Hormones	Heme	*HMOX1*	ORF3a	Y	Y
Degradation	Hormones	melatonin	*POR*	Nsp2	Y	Y
Degradation	Hormones	Adrenalin	*COMT*	Nsp2	Y	Y
Degradation	Hormones	L-dopa degradation	*COMT*	Nsp2	Y	Y

Our results identify host dependency factors required for the SARS-CoV-2 virus infection, replication, survival, and viability within different cell compartments and provide insight into novel treatment strategies.

### Essential reactions for the host and viral metabolism

The Flux Balance Analysis (FBA) method (Orth et al, 2010) was used to compute both the biomass maintenance of the cell in the absence of virus and the maximum growth rate of the virus in the cell (host optimum and virus optimum conditions). We identified 52 essential reactions in the macrophage (*i*AB-AMØ-1410) model and 10 reactions in the epithelial cell model (*i*HsaEC21) essential for the virus to propagate (Tables S1 and S2). It was also demonstrated that: (i) the maximal biomass maintenance of the macrophage cell in the absence of virus was 0.0269 (Table S1) and 0.012 h^−1^ for the human airway epithelial cell (Table S2); (ii) the maximum growth rate of the virus in the macrophage cell was 0.0144 and 0.0181 h^−1^ in the human airway epithelial cell. These numerical results mean that 0.0144 h^−1^ is the theoretical maximum of the growth rate of the virus in the human macrophage cell. If this flux is assigned to the viral growth reaction, then flux variability analysis (FVA) (Orth et al, 2010) can be used to calculate the ranges of fluxes allowed for the remaining reactions in the cell while the virus is being replicated at its optimum condition. The execution of FVA under such conditions produced zero biomass maintenance of the host cell, that is, both the lower and upper flux bounds of the reaction indicate that the growth is zero. This means that if the virus is replicating at its maximum rate, then the cell will not be viable.

Table S1 List of essential reactions in the Macrophage model (*i*AB-AMØ-1410).

Table S2 List of essential reactions in the Human Airway epithelial model (*i*HsaEC21).

### Bottleneck reactions and the prioritization of potential drug targets

The bottleneck reactions identified by the *findCPcli* tool are unique reactions of the metabolic network required for the growth and survival of the organism and, like chokepoint reactions, are potential drug targets (Yeh et al, 2004; Oarga et al, 2020). Although classical chokepoint reactions identify reactions that are the only producers or the only consumers of a given metabolite and consider just the model structure, we improve on this approach by using both the structural and dynamical information. FVA is used to compute flux bounds of the reactions, and in turn, to determine whether a given reaction is reversible or not. Reversibility will be used to obtain the sets of metabolites that can be produced and consumed by the reactions, and thus, to compute flux-dependent bottleneck reactions. This approach has been applied to the integrated Human/SARS-CoV-2 metabolic model within the airway epithelial cell and the macrophage cell to predict potential drug targets against SARS-CoV-2.

We initially identified 1,595 bottleneck reactions required for the virus’ maintenance and replication in the *human macrophage* cell; these include pathways in lipid metabolism, coenzyme transport and metabolism, energy production and conversion, and amino acid and nucleotide transport and metabolism (Table S3). In the human airway epithelial cell, 1,598 bottleneck reactions were initially identified; these include the biosynthesis and degradation pathways of amino acids, fatty acids, carbohydrates, amines, cofactors, and some components of the central mRNA metabolism (Table S4).

Table S3 List of bottleneck reactions in the Macrophage model (*i*AB-AMØ-1410).

Table S4 List of bottleneck reactions in the Human Airway epithelial model (*i*HsaEC21).

Because each bottleneck reaction should be balanced by at least one other reaction that produces or consumes that metabolite, we have excluded reactions in the model with dead-end metabolites. The bottleneck reactions are potential drug targets as they are indispensable for the maintenance and replication of the virus within the host. To rank the potential drug targets identified, we prioritised enzymes for unique reactions that occur at the nodes of intersection between the bottleneck and essential reactions and the results from the human/virus protein–protein interaction network (Gordon et al, 2020) (Table 1). The following steps were taken to label the reactions which occur at the nodes of intersections between the referenced points above: (1) we initially identified both the bottleneck and essential reactions to the virus within the model, (2) we highlighted the interactions between the viral proteins and the human metabolic enzymes, and (3) we selected the reactions from the human/virus interactions which are also present in the list of bottleneck and/or essential reactions.

The enzymes of the reactions at the nodes of intersection are (1) host dependency factors identified by the model as necessary for the survival of the virus and (2) proteins from experimental datasets with high-confidence virus–human protein–protein interaction data; we refer to these points of intersection as PPi-pathway intersection nodes.

**Figure 3.**
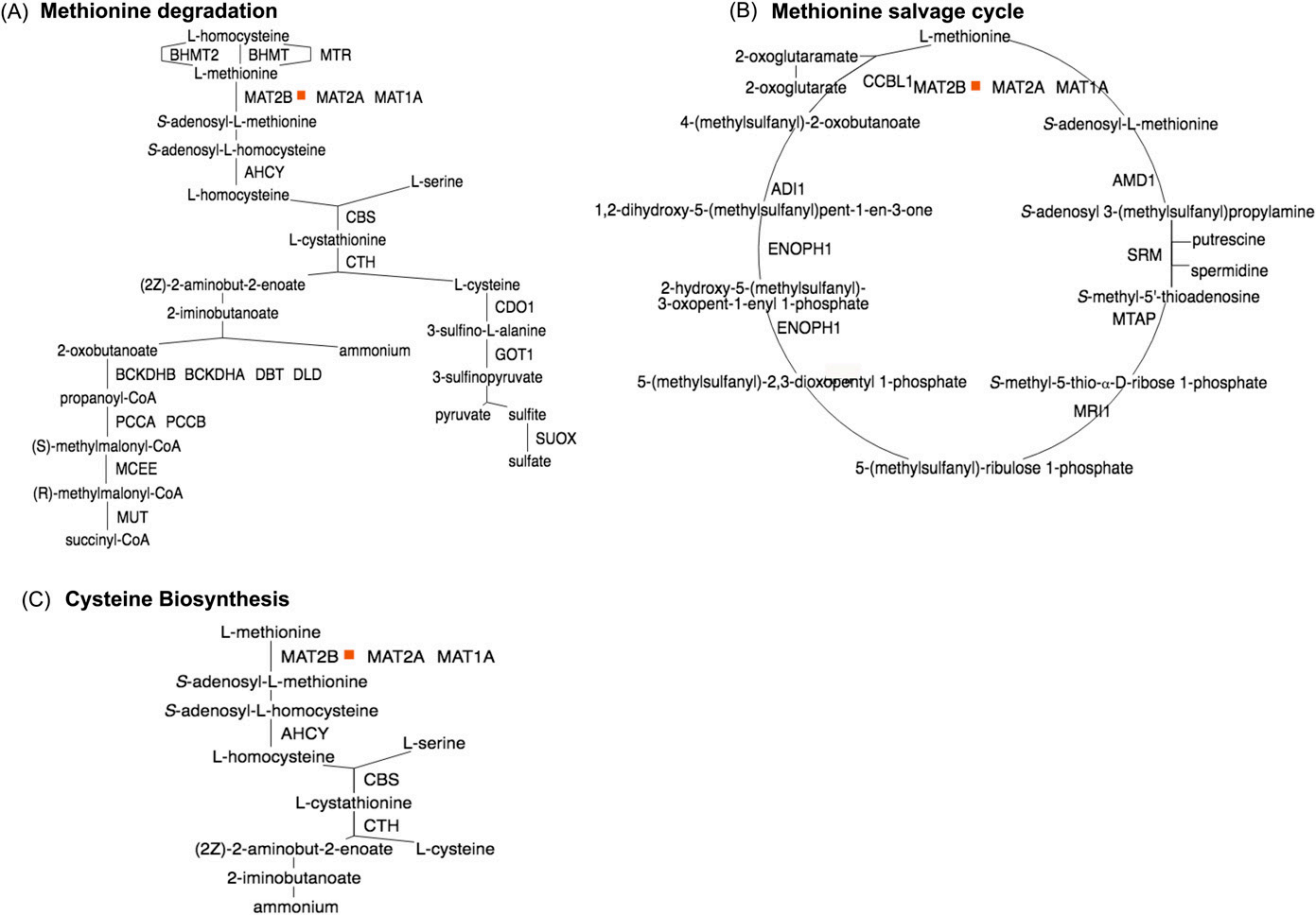
PPi-pathway intersection node—Nsp9. **(A)** Methionine degradation. **(B)** Methionine salvage cycle. **(C)** Cysteine biosynthesis.

**Figure 4.**
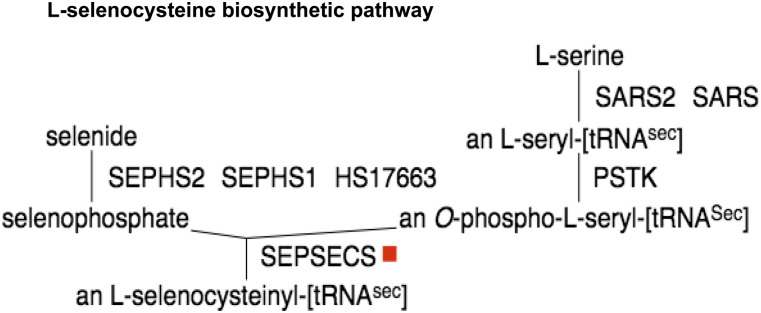
PPi-pathway intersection node—Nsp8.

The PPi-pathway intersection (P-Pi) nodes identified are present in biosynthesis pathways such as the cysteine and S-adenosyl-L-methionine biosynthetic pathways. In both pathways, the enzyme S-adenosylmethionine synthase (*MAT2B*) catalyses the phosphorylation reaction of methionine to S-adenosyl-L-methionine. During infection, the viral protein Nsp9 is seen to react with *MAT2B* (Gordon et al, 2020) (Fig 3A–C). Another viral protein, Nsp8, also interacts with the enzyme O-phosphoseryl-tRNA(Sec) selenium transferase (*SEPSECS*), which catalyses the last step of the L-selenocysteine biosynthesis pathway (Fig 4).

**Figure 5.**
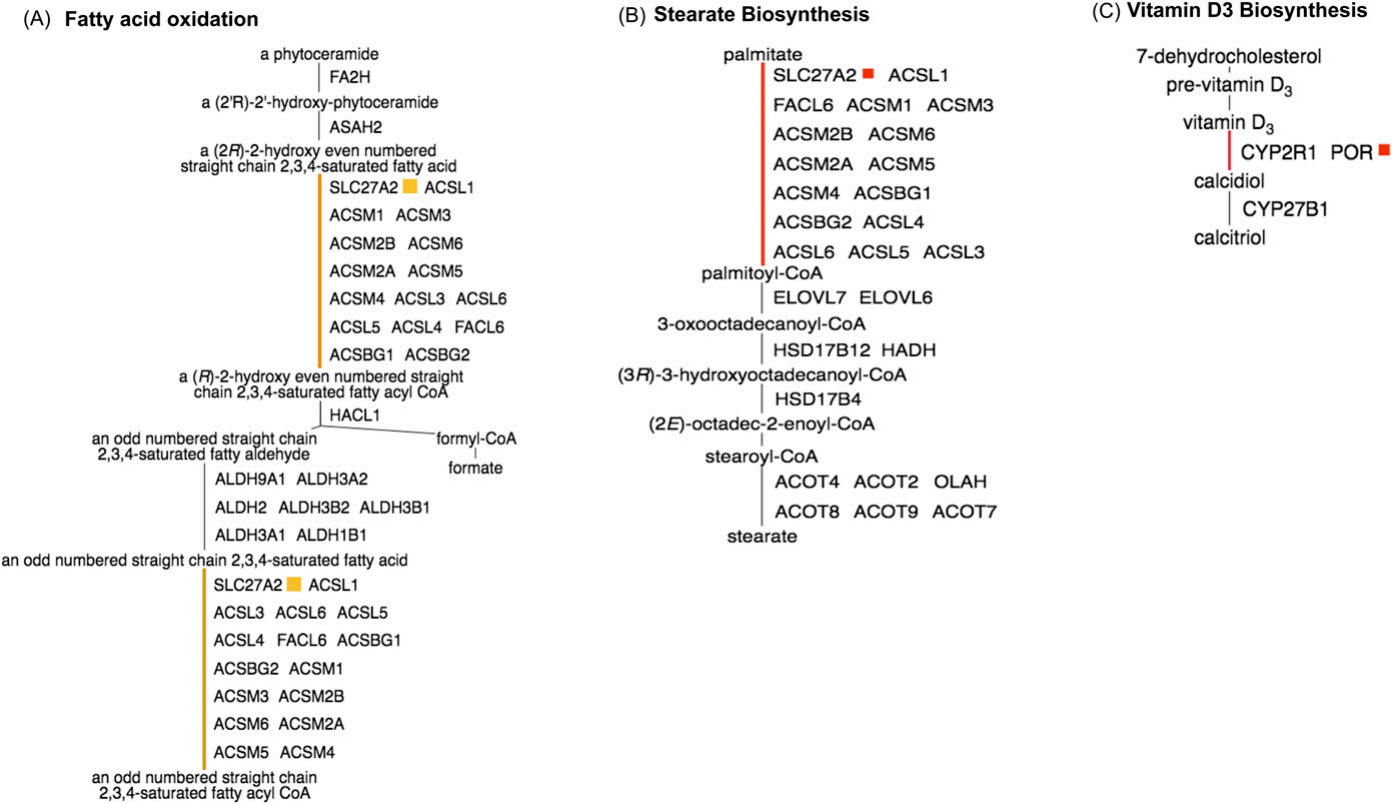
PPi-pathway intersection node—Nsp2. **(A)** Fatty acid oxidation. **(B)** Stearate biosynthesis. **(C)** Vitamin D3 biosynthesis.

P-Pi nodes also occur in a network of various fatty acid and stearate biosynthetic pathways with Nsp2 interacting with the very long-chain acyl-CoA synthetase (*SLC27A2*) (Fig 5A and B). The viral protein, Nsp2, also interacts with *POR* in other pathways including vitamin D3 biosynthesis (Fig 5C) and in L-tryptophan degradation, ceramide, and sphingolipid recycling pathways (Table 1).

**Figure 6.**
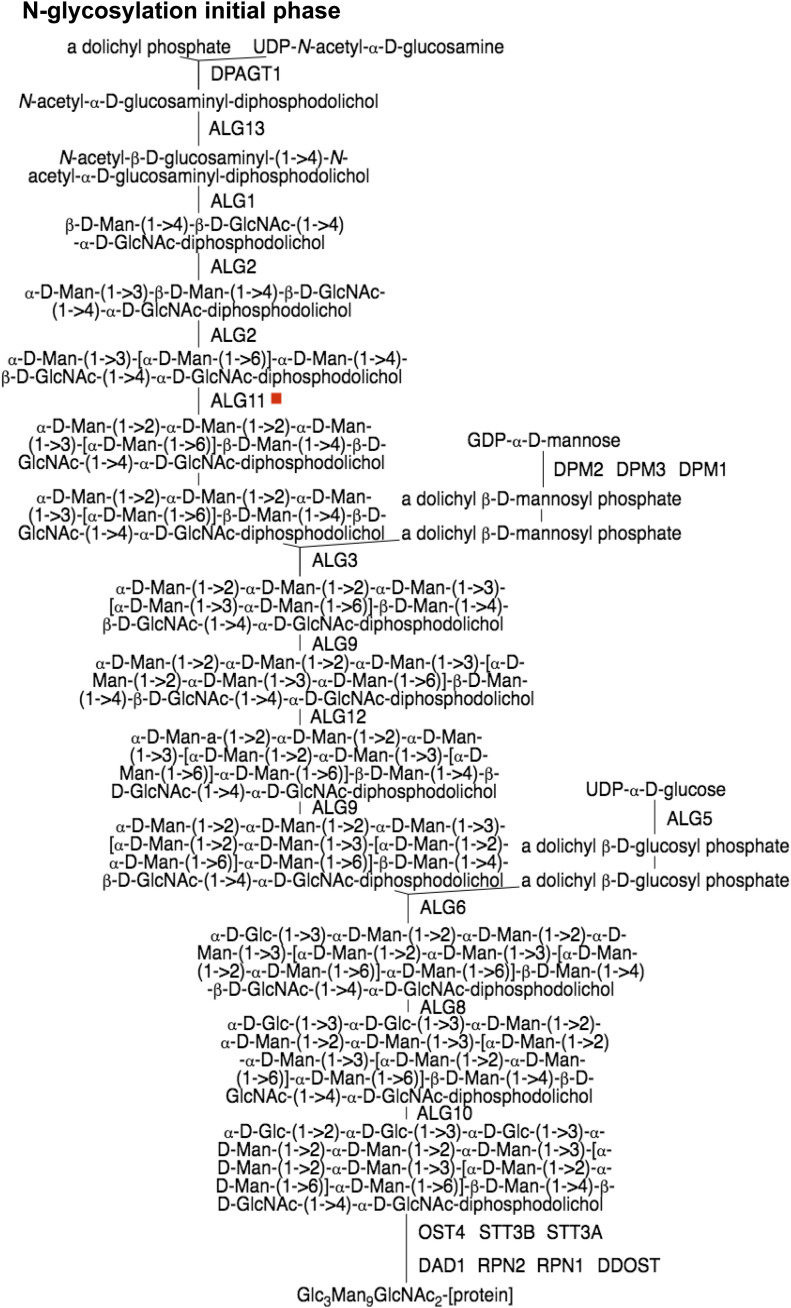
PPi-pathway intersection node—Nsp4.

**Figure 7.**
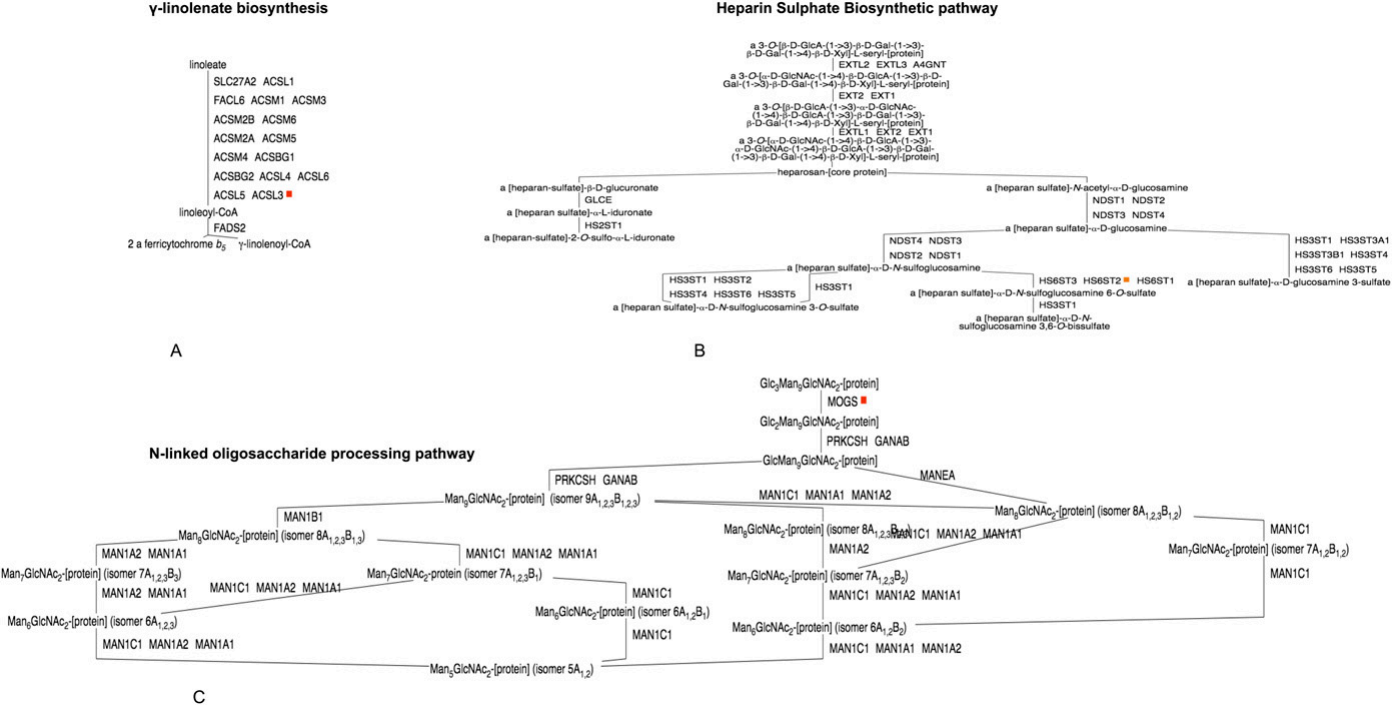
Pi-pathway intersection nodes - Nsp7 and Orf8a in carbohydrate and fatty acid metabolism. **(A)** PPi-pathway intersection node—Nsp7. **(B)** PPi-Pathway intersection node—Orf8a. **(C)** PPi-pathway intersection node—Nsp7 and Orf8a.

In carbohydrate metabolism, a P-Pi node is identified at the glycan and oligosaccharide biosynthetic pathways, and specifically where two mannose residues are added in α (1→2) linkages to the nascent oligosaccharide and catalysed by the enzyme *ALG11*. The viral protein Nsp4 interacts with *ALG11* during the infection of the SARS-CoV-2 virus (Fig 6). Another viral protein, Nsp7, interacts with *ACSL3* in the γ-linolenate biosynthesis (Fig 7A). Nsp7 also reacts with *ACSL3* and ORF8a interacts with *HS2ST1* (Fig 7B), a key enzyme involved in the heparan sulfate biosynthesis pathway. The first enzyme of the N-linked oligosaccharide processing pathway, mannosyl-oligosaccharide α-1,2-glucosidase (*MOGS*), also interacts with Nsp7 and ORF8a (Fig 7C).

P-Pi nodes specific to the human macrophage cell include the O-phosphoseryl-tRNA(Sec) selenium transferase in the L-selenocysteine biosynthetic pathway, which interacts with the viral protein Nsp8. The alkylglycerone-phosphate synthase/Nps7 P-Pi node, which is present in the phospholipid/plasmalogen biosynthetic pathway is also specific to the macrophage cell. Alternatively, PPi-pathway intersection nodes common to both human airway epithelial cell and the macrophage cell include the *MAT2B*/Nsp9 intersection pathways present in the cysteine metabolism and L-methionine degradation. P-Pi nodes specific to the human epithelial cell include the peptidyl-glutamate 4-carboxylase present in the Vitamin K epoxide cycle and the α-aminoadipic semialdehyde synthase enzyme in the L-lysine degradation pathway.

## Discussion

Metabolic pathway perturbations in the human cell due to COVID-19 reflect the viral entry and infection of SARS-CoV-2 and the immune regulation changes in the human body. We have used in silico models to study the interactions of SARS-CoV-2 in the host and propose new treatment management regimens. We have built on studies using the human alveolar macrophage model *i*AB-AMØ-1410 (Bordbar et al, 2010) as host cells and SARS-CoV-2 (Renz et al, 2020, 2021); influenza ((Aller et al 2018)), and tuberculosis as pathogens and developed a new integrated model of the human epithelial cell and SARS-CoV-2.

The initial draft epithelial cell model was based on the human Recon1 model and consists of 1,206 reactions, 994 metabolites, and 0 genes (Wang et al, 2012). Our revised *i*HsaEC21 epithelial cell model contains 4,660 genes, 3,614 reactions, and 4,052 metabolites initially scored 34% on MEMOTE (Lieven et al, 2020). After further refinements using Recon3D, HumanCyc, MetaNetX 4.2, BiGG, ChEBI, MetaCyc, and PubChem databases (Caspi et al, 2014; Hastings et al, 2016; Norsigian et al, 2020; Moretti et al, 2021) including manual curations with literature, *i*HsaEC21 now has a MEMOTE quality score of 51% (Supplemental Data 1). Although the *i*AB-AMØ-1410 (Bordbar et al, 2010) was also developed from the human Recon1 model, there was further refinement with literature, which attributed to its quality score of 45% (Supplemental Data 2) compared with the draft epithelial cell model from Wang et al (2012) which had a low-quality score of 19% (Supplemental Data 3).

Supplemental Data 2. MEMOTE results of the integrated macrophage model (iAB_AMO1410_SARS-CoV-2) and SARS-CoV-2.

Supplemental Data 3. MEMOTE results of the draft human airway epithelial model by Wang et al (2012).

Previous studies have also demonstrated the role of the ACE2 as the receptor for both the SARS-CoV and the SARS-CoV-2. The ACE2 cells are expressed in the human airway epithelial cells (Hamming et al, 2004; Liu et al, 2020). In this study, we have constructed an integrated human epithelial cell and SARS-CoV-2 to provide insight into the infection patterns of the virus in the human body.

Our in silico comparative analyses of the SARS-CoV-2 viral infection between two different conditions (infected human macrophage and airway epithelial cells) show the requirements of viability of the virus between these two conditions. Our results complement previous efforts to propose drug targets and repurposing strategies including the SARS-CoV-2-Human Protein–Protein Interaction Map Gordon et al, (2020 and studies from Steward, 2020, which identified host dependency factors facilitating virus infection. We also provide additional resources to the COVID-19 disease map (Ostaszewski et al, 2020). In addition, we have designed a new algorithm (Oarga et al, 2020) and used the dynamic information of the human/virus models to predict new treatment regimens. Here, we demonstrate the flux changes from lipid metabolism, cofactor biosynthetic pathways, redox balance, and immune regulation indicative of pathogenic reactions arising from the COVID-19 viral infections.

### Inhibition of viral entry and replication

The components of the plasma membrane, such as cholesterol and sphingolipid-rich lipid, Abu-Farha et al, (2020) are involved in virus penetration, entry, replication, and infection (Wang et al, 2008; Abu-Farha et al, 2020). In this study, we demonstrate current therapeutic strategies that interfere with different stages of the viral cycle by targeting lipid metabolism and proposing new treatment strategies.

The dynamical changes of flux metabolism in our in-silico virus optimal models show a significant increase in viral infection. Four reactions involved in the biosynthesis of fatty acids with predicted non-zero fluxes in the host model exhibit an average increase of 190% in their maximum fluxes in the viral model (the maximum increase is 298%). The average increase of 32 reactions in lipid metabolism with non-zero fluxes in the host model is 277% (the maximum increase is 498%). Concerning sphingolipid metabolism, 14 of 15 reactions with non-zero fluxes in the host model exhibit an average increase of 228% (the maximum increase is 298%) and similar increases in phospholipases and palmitic acid biosynthesis (Fig 5A and B). We show an average increase of 190% in cholesterol and fatty acid metabolism during viral infection and demonstrate the essentiality of these pathways to SARS-CoV-2. Previous studies have shown that cholesterol and fatty acids are main components of the viral membranes and needed for viral replication (Heaton & Randall, 2011); therefore, drugs inhibiting these pathways such as AM580, statins, and fibrate (Fiévet & Staels, 2009) will be essential for both early and late stages of COVID-19.

Sphingolipids are composed of both hydrophobic and hydrophilic units and play a large role in the endocytic or exocytic viral entry processes into the cell (Dimitrov, 2004). The pH-dependent endocytic process is further enhanced by the presence of clathrin, a protein present in the plasma membrane, the Golgi apparatus and in the cytoplasm, whereas the exocytic route involves viral crossing through the plasma membrane at neutral pH. Our results show a threefold increase in sphingolipid metabolism during viral infection (Fig 5); we hypothesise that drugs inhibiting sphingolipid metabolism and/or the endocytosis process will inhibit infection of SARS-CoV-2. Indeed, a sphingosine kinase-2 (SphK2) inhibitor, opaganib, which has proved beneficial in the treatment of COVID-19, is currently in global phase 2/3 clinical trials and in US phase 2 studies. Previous studies also demonstrate that chloroquine and hydroxychloroquine elevate the pH of endosomes in the cells and directly inhibit endocytosis and the exocytic process (Munro et al, 1997; Devaux et al, 2020). More recent studies have also shown that artemisinin-inhibited endocytosis (Hoppe et al, 2004; Uzun & Toptas, 2020). A recent study by Abu-Farha described other lipid-modifying drugs including LJ-001, arbidol, methyl-B-cyclodextrin (Blaising et al, 2013; de Wilde et al, 2014; Mazzon & Marsh, 2019). We highlight critical reactions as drug targets for lipid metabolism for the SARS-CoV-2 virus (Table S2) and in our PPi-pathway intersection nodes (Table 1).

Our lists of bottleneck, essential reactions, and PPi-pathway intersection nodes also include critical points in the biosynthesis of phospholipids. We show that these reactions are essential for viral infection and replication (Table 1) and propose targeting the phospholipase enzyme or the interacting Nsp2 protein to inhibit viral replication. Our results support previous studies from Müller et al (2018) that targeting the phospholipase enzyme could inhibit the early stage of COVID-19.

### Redox homeostasis and antioxidant therapy

Redox homeostasis refers to the ability of the cell to maintain its balance amidst infections and other unstable cellular environmental factors. Delgado-Roche and Mesta (2020) have described oxidative stress as a key player in severe acute respiratory syndrome coronavirus (SARS-CoV) infection with cytokine production. Foyer and Noctor (2005) have previously shown that antioxidants, such as glutathione and ascorbate, are important metabolites for the cellular redox state. Our studies have identified key target enzymes involved in the metabolism of glutathione and ascorbic acid as bottleneck and essential reactions, including glutathione synthase, glutathione peroxidase, and ascorbic acid oxidase (Table S2). We also demonstrate an increase in the flux of these enzymatic reactions on infection of the virus. In a recent study, Horowitz et al (2020) previously demonstrated how the use of high dose oral and/or IV glutathione on severe outcomes of SARS-CoV-2 led to favourable treatment outcomes. Other studies have shown that steroids such as dexamethasone and Methylprednisolone are used to treat severe cases of COVID-19. Because of the possible side effects of steroid treatment, we propose the use of glutathione as therapy for severe cases of COVID-19 in the aged population and other severe cases with cytokine storm syndrome.

### Immune regulation

SARS-CoV-2 can proliferate unhindered in infected cells because of the lack of immunity in humans (Felsenstein et al, 2020). The result is cell death, a release of viral particles to the extracellular environment and a general hyperactivity of the immune system in some patients with severe COVID-19 disease and subsequent lung inflammation and cytokine syndrome. Immunocompromised patients or those with underlying symptoms such as diabetes, hypertension, and transplantation are most affected (Zhong et al, 2020). Although clinical trials are ongoing worldwide with various antivirals and immune-modulating treatments, there is currently limited knowledge on the host dependency factors responsible for the individual outcomes of the disease. Our results provide insight into the immune evasion strategies of the SARS-CoV-2 virus; we demonstrate changes in the flux metabolism of vitamin D and tryptophan metabolism during viral infection. Vitamin D is important for bone growth and turnover and a low vitamin D status is associated with increased susceptibility to upper respiratory tract infections (Mitchell, 2020). Previous studies have shown that supplementation of vitamin D prevents acute respiratory tract infections (Martineau et al, 2017). Our results highlight vitamin D as an essential reaction in the PPi-pathway intersection nodes and we show the viral protein Nsp2 interaction with key enzymes in the vitamins D and C metabolism pathways (Fig 5C). SARS-CoV-2 viral infection causes metabolic perturbations of vitamin D metabolism in the host resulting in disruptions in cellular homeostasis. We propose support therapy management strategies where vitamin D supplements are provided to all COVID-19 patients. Our results also show that tryptophan, melatonin and prostaglandins, important compounds for immunity and homeostasis (Gitto et al, 2011; Platten et al, 2019), are affected by the infection of SARS-CoV-2 and we provide insight into the viral mechanism of action within the human body.

In summary, we have provided a platform for drug target prediction against COVID-19, and future studies on viral entry inhibition, antioxidant therapy, and immune regulation.

## Materials and Methods

For our modelling analyses, we based our reconstruction in the integrated macrophage cell with the SARS-CoV-2 virus model, previously constructed by Renz et al (2020) to develop a new human airway epithelial cell integrated with the SARS-CoV-2 virus. We predicted the reactions which were critical for the survival of the virus in both cell compartments (macrophage and epithelial cells); our results provide insights into COVID-19 treatment management strategies.

### GEM reconstruction, curation, and simulations

**Figure 8.**
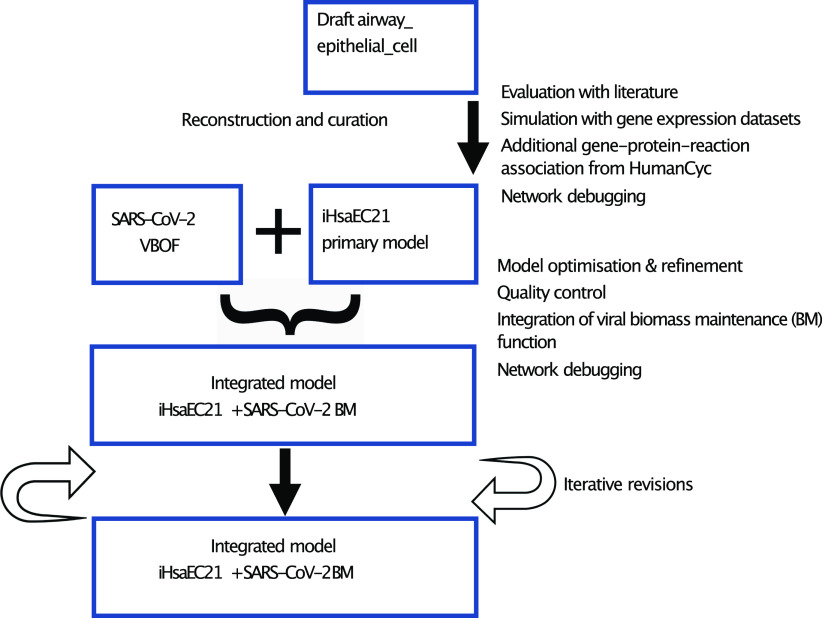
Development of an integrated model of the Human Airway Epithelial cell and SARS-CoV-2.

We obtained an automated draft reconstruction of the human airway epithelial cell (Wang et al, 2012) and evaluated the metabolic functions and reactions collected in the draft reconstruction against organism-specific literature. We used the gene expression datasets of the human airway epithelial cell (Deprez et al, 2019 *Preprint*; Vieira Braga et al, 2019) to curate, annotate and improve on the model (Fig 8). We manipulated and simulated the model, including the addition of the gene expression data, with COBRApy (Ebrahim et al, 2013) and GLPK (https://www.gnu.org/software/glpk/). We obtained additional reactions, gene-to-reaction associations, and pathways (that were not in the automated model) from HumanCyc (Trupp et al, 2010) to develop a new genome-scale metabolic model (GEM) of the human airway epithelial cell. We improved on the new reconstruction by mapping the genes and reactions of the GEM to Recon3D, a standard human metabolic model, MetaNetX 4.2, BiGG, ChEBI, MetaCyc, and PubChem databases (Caspi et al, 2014; Hastings et al, 2016; Norsigian et al, 2020; Moretti et al, 2021) and produced a revised human airway epithelial cell *i*HsaEC21 (Fig 8). The revised model reconstruction, *i*HsaEC21, can be instantiated without error on the COBRA software (version 0.16.0) (Ebrahim et al, 2013).

### Model optimisation

To improve on the quality of the model, we removed the reactions with dead-end metabolites, which were previously found in the automated model. This was followed by iteratively assessing the model and accounting for the cell-specific metabolic and exchange reactions in the epithelial cell model. We compared and performed various iterations of the new GEM with Recon3D, MetaCyc, MetaNetX 4.2, BiGG, ChEBI, and PubChem databases (Caspi et al, 2014; Hastings et al, 2016; Norsigian et al, 2020; Kim et al, 2021; Moretti et al, 2021). As a result, we added charge and formulae to 1,473 compounds on the new GEM. During the iterations and optimisation of the model, we performed quality control checks on MEMOTE, a standardised genome-scale metabolic model testing software (Leiven et al, 2020).

### Integration of the human airway epithelial cell model and the SARS-CoV-2 virus

We integrated the viral biomass maintenance function, previously developed for the macrophage cell (Renz et al. 2020, 2021) into the new GEM to produce an integrated model of *i*HsaEC21+SARS-CoV-2. We interrogated the new model to identify the host-dependency factors for the SARS-CoV-2 virus by using the novel software tool *findCPcli*.

The new GEM is encoded in the Systems Biology Markup Language (SBML) (Keating et al, 2020) (*i*HsaEC21); i for in silico, Hsa for *H. sapiens*, and EC for airway epithelial cell published in 2021. *i*HsaEC21 consists of 3,752 reactions, 3,914 metabolites, 4,660 genes, and 48 metabolic pathways.

To assess and predict the performance of the models, we made use of FBA and FVA (Orth et al, 2010). FBA is a computational method that can be applied efficiently to genome-scale models to estimate the fluxes of reactions at a steady state. It is based on the solution of a linear programming problem that maximizes an objective function of interest subject to a set of constraints on the fluxes of the reactions. The linear programming problem associated with FBA can be expressed as follows:

maxc·v,

s.t. S·v=0,

L ≤ v ≤ U,

where *v* is the vector of fluxes, *c* represents the objective function coefficients, *S* is the stoichiometry matrix, and *L* and *U* are lower and upper bounds on the fluxes. Thus, *c v* is the objective function, which usually refers to the growth rate of the organism and *S v* = 0 represents the balance of fluxes at steady state.

FVA is also based on the solution of linear programming problems, and its main use is the computation of ranges of fluxes that are compatible with given flux constraints. For instance, if the growth rate predicted by FBA is μ_max_, then the range of fluxes of a given reaction *i* that are compatible with such growth rate can be obtained by minimizing and maximizing the following programming problem:

$$\min/\max\;v_{i},$$

s.t. S·v=0,

L ≤ v ≤ U,

$$v_{\mathrm{growth}}=\mu_{\max},$$

where *v*_*growth*_ is the flux of the reaction associated with growth and *v*_*i*_ is the flux of reaction *i*. FBA and FVA were computed on the metabolic network of the host, both with and without the reaction modelling the production of the virus, by using the Python toolbox COBRApy.

Bottleneck reactions, like chokepoint reactions, are required for the reaction synthesis and the removal of these reactions will cause an accumulation or depletion of the metabolites; thus, they represent potential drug targets. The software tool *findCPcli* was developed to compute bottleneck reactions on genome-scale models by considering the structural and dynamic information of the models. The dynamic information is considered as follows: (a) FVA is run to compute lower and upper flux bounds of the reactions; (b) the obtained flux bounds are used to identify reversible and non-reversible; and (c) this directionality of reactions is used to determine consumer and producer reactions, and in turn, bottleneck reactions. In addition to the computation of bottleneck reactions, *findCPcli* can also compute and remove dead-end metabolites, find essential reactions, and update the flux bounds of the reactions according to the results of FVA.

## Data Availability

The model has been deposited as an SBML Level 3 Version 1 (Hucka et al, 2018) file with FBC extension and the minimal information required in the annotation of models (MIRIAM) (Juty et al, 2012) in BioModels (https://www.ebi.ac.uk/biomodels/MODEL2007210001) (Malik-Sheriff et al, 2020). The source code of *findCPcli* together with its documentation is available at https://github.com/findCP/findCPcli. The tool requires Python 3.5 (or higher) and can be installed with *pip*, the standard package installer for Python. The pathway maps were created with the pathway collage software (Paley et al, 2016) and the essential reactions are available as a smart table on HumanCyc (https://biocyc.org/group?id=biocyc17-29351-3833343490).

## Supplementary Material

Reviewer comments
